# Effects of multimodal prehabilitation on surgery outcomes: prospective stepped-wedge, hospital-wide implementation study

**DOI:** 10.1093/bjs/znag013

**Published:** 2026-02-17

**Authors:** Luuk D Drager, Femke Atsma, Dieuwke Strijker, Linda A G van Heusden-Scholtalbers, Monique J M D van Asseldonk, Jonas Rosenstok, Joost P H Seeger, Sjors Verlaan, Laurien M Buffart, Cornelis J H M van Laarhoven, Baukje van den Heuvel, the F4S PREHAB Collaborative Group, K Allewijn, M G A van den Berg, P B van den Boezem, J J Bonenkamp, A J A Bremers, M Dirven, S A W van de Groes, M Groos, A G van der Heijden, J Honings, H Jager-Wittenaar, B R Klarenbeek, J M van Koeveringe, B M van der Kolk, M ter Laan, S Muselaers, J M A Pijnenborg, P Servaes, D Smits, S Teerenstra, T Verhoeven, J H W de Wilt

**Affiliations:** Department of Surgery, Radboud University Medical Centre, Nijmegen, the Netherlands; IQ Health Science Department, Radboud University Medical Centre, Nijmegen, the Netherlands; Department of Surgery, Radboud University Medical Centre, Nijmegen, the Netherlands; Department of Rehabilitation, Radboud University Medical Centre, Nijmegen, the Netherlands; Department of Gastroenterology and Hepatology, Dietetics, Radboud University Medical Centre, Nijmegen, the Netherlands; Department of Surgery, Radboud University Medical Centre, Nijmegen, the Netherlands; Department of Healthcare and Services, HAN University of Applied Sciences, Nijmegen, the Netherlands; Department of Surgery, Radboud University Medical Centre, Nijmegen, the Netherlands; Department of Nutrition and Dietetics, Faculty of Health, Sport and Physical Activity, Amsterdam University of Applied Sciences, Amsterdam, the Netherlands; Department of Medical Biosciences, Radboud University Medical Centre, Nijmegen, the Netherlands; Department of Surgery, Radboud University Medical Centre, Nijmegen, the Netherlands; Department of Surgery, Radboud University Medical Centre, Nijmegen, the Netherlands

## Abstract

**Background:**

Multimodal prehabilitation may improve surgical outcomes in selected populations, but its real-world effectiveness remains unclear. The aim of this study was to evaluate the effect of hospital-wide implementation of multimodal prehabilitation on postoperative complications and length of hospital stay in a diverse surgical population.

**Methods:**

This single-centre, non-randomized stepped-wedge study (F4S PREHAB) was conducted at Radboudumc, Nijmegen, The Netherlands from March 2019 to April 2024. Patients who underwent elective surgery across 20 clinical pathways received either standard preoperative care or multimodal prehabilitation comprising supervised exercise, nutritional support, psychological counselling, and smoking and alcohol cessation support. The primary outcome was the incidence and severity of postoperative complications within 30 days, with assessment of Clavien–Dindo (CD) grade ≥II complications and the dichotomized Comprehensive Complication Index (CCI). The secondary outcome was the length of hospital stay. Analyses used generalized linear models adjusted for time of inclusion and clinical pathway, as well as other confounders in some models. Subgroup analyses focused on patients who underwent high-risk gastrointestinal (GI) oncological surgery.

**Results:**

During the study interval, 4131 patients received usual care (2660 patients) or prehabilitation (1471 patients). A total of 367 patients (24.9%) attended at least nine exercise sessions, indicating partial adherence. No significant differences between groups were found with regard to postoperative CD grade ≥II complications (adjusted risk ratio 1.02 (95% c.i. 0.90 to 1.16)) and CCI >22.6 (adjusted risk ratio 1.03 (95% c.i. 0.86 to 1.23)) or length of hospital stay (adjusted incidence rate ratio 1.04 (95% c.i. 0.92 to 1.18)). In the high-risk GI oncological surgery subgroup (1230 patients), the relative reduction in CD grade ≥II complication risk was 9%, but this was not statistically significant (adjusted risk ratio 0.91 (95% c.i. 0.75 to 1.10)).

**Conclusion:**

Hospital-wide implementation of multimodal prehabilitation did not reduce postoperative complications or length of hospital stay. A greater effect in high-risk patients suggests a targeted approach may be more effective. Future research should identify such patients and evaluate effectiveness of prehabilitation in this population.

## Teaser

In this hospital-wide stepped implementation study, multimodal prehabilitation showed partial adherence and did not reduce postoperative Clavien–Dindo grade ≥II complications or length of hospital stay in a heterogeneous surgical population. Although no statistically significant effects were observed, the confidence intervals included potentially clinically meaningful differences, meaning a relevant effect cannot be excluded. Exploratory analyses suggested a trend towards fewer complications in patients undergoing gastrointestinal oncological surgery, supporting further evaluation of targeted prehabilitation in high-risk populations.

## Introduction

Surgical intervention remains a cornerstone of treatment across multiple medical specialties, including oncology, vascular disease, and orthopaedics. However, surgery imposes substantial physiological and functional strain through tissue injury, the systemic stress response, and postoperative immobility^1^. Despite advances in perioperative care such as minimally invasive techniques and enhanced recovery after surgery (ERAS), >20% of patients experience postoperative complications, varying by type of surgery and preoperative risk factors^2,3^. These complications contribute to prolonged hospital stays, increased mortality, functional decline, and reduced quality of life^4^  ^,5^.

Preoperative risk factors include advanced age, frailty, low physical fitness, poor nutritional status, and psychological distress^6^. As chronic disease becomes more prevalent and healthcare systems face increasing pressure, there is rising interest in enhancing patients’ physical fitness and health before surgery through multimodal prehabilitation^7–9^. This approach combines supervised physical exercise, nutritional support, psychological counselling and support, and aid in smoking and alcohol cessation.

Multimodal prehabilitation has most frequently been investigated in colorectal cancer (CRC) surgery, showing favourable effects on both postoperative complications and functional recovery^10,11^. Although the physiological mechanisms targeted by prehabilitation may be shared across different patient groups, the generalizability of findings to other surgical populations remains uncertain. In addition, much of the existing evidence is derived from RCTs, which have been characterized by substantial heterogeneity in intervention components, duration, and outcome measures, resulting in variable findings and an overall low certainty of evidence^12^. Consequently, extrapolation of RCT results to routine clinical practice is limited^13^. This, together with the increasing demands on healthcare capacity, highlights the need for scalable, pragmatic studies assessing the effectiveness of prehabilitation in routine clinical practice across diverse surgical populations in a real-world setting.

To address these challenges, the hospital-wide stepped-wedge F4S PREHAB study was conducted to evaluate the effect of a multimodal prehabilitation programme on complication risk and length of hospital stay across 20 surgical clinical pathways, encompassing a heterogeneous population undergoing elective surgical procedures.

## Methods

### Study ethics

The study protocol was published previously^14^ and was approved by the local Medical Ethics Committee (METC Oost-Nederland; NL73777.091.20). Written informed consent was obtained before study participation. The study was registered under study ID NL-OMON28368 (https://onderzoekmetmensen.nl/nl/trial/28368).

### Study design

**Fig. 1:**
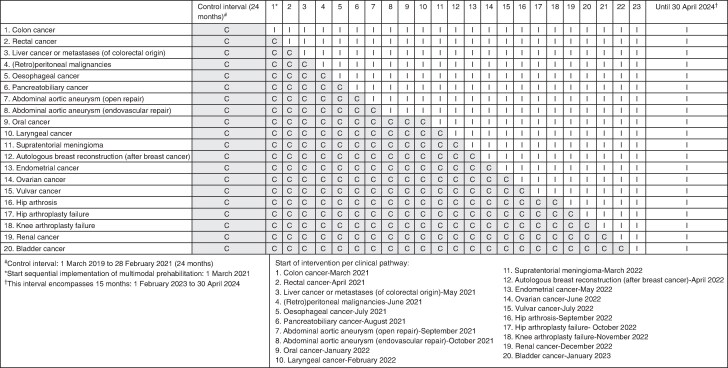
Implementation timeline of clinical pathway transitions C, control; I, intervention.

The F4S PREHAB study is a single-centre stepped-wedge study comparing standard preoperative care with multimodal prehabilitation at Radboudumc, Nijmegen, The Netherlands. In the design, the steps consisted of 20 predefined clinical pathways, which transitioned sequentially from standard care to multimodal prehabilitation. Although randomized implementation was considered during study planning, the order of implementation was ultimately determined by logistical feasibility and pathway readiness rather than randomization given the scale and complexity of hospital-wide implementation. Prehabilitation was introduced pathway by pathway, one clinical pathway per month (*Fig. 1*). Eligible patients received either standard preoperative care or multimodal prehabilitation as part of their preoperative treatment. Patients were informed about the study procedures by a member of their clinical care team and subsequently referred for an intake and baseline assessment.

### Participants

Patient inclusion and data collection occurred between 1 March 2019 and 30 April 2024 (*Fig. 1*). For efficiency reasons and to increase power, the start of the control interval was 24 months before the actual implementation and start of the prospective part of the study. In this interval, data from all eligible patients were retrospectively retrieved from medical records. From 1 March 2021 to 30 April 2024, stepwise implementation of multimodal prehabilitation across clinical pathways was conducted and data were prospectively collected from medical records.

Adult patients who underwent elective surgery for a diagnosis included in one of the 20 defined clinical pathways (*Supplementary material*) were eligible for inclusion. These clinical pathways comprised: colon cancer, rectal cancer, liver cancer or metastases (of colorectal origin), (retro)peritoneal malignancies, oesophageal cancer, pancreatobiliary cancer, abdominal aortic aneurysm (open repair), abdominal aortic aneurysm (endovascular repair), oral cancer, laryngeal cancer, supratentorial meningioma, autologous breast reconstruction (after breast cancer), endometrial cancer, ovarian cancer, vulvar cancer, hip arthrosis, hip arthroplasty failure, knee arthroplasty failure, renal cancer, and bladder cancer. Patients were excluded if they had an ASA grade of ≥IV, were unable to read or understand the Dutch language, or had a contraindication to protein supplementation or high-intensity exercise according to the guidelines of the American College of Sports Medicine^15^.

A gastrointestinal (GI) oncological surgery subgroup (that is patients with colon cancer, rectal cancer, liver cancer or metastases (of colorectal origin), (retro)peritoneal malignancies, oesophageal cancer, and pancreatobiliary cancer) was predefined, because such patients were considered to be at high risk of postoperative complications.

### Intervention group

Participants followed a tailored programme designed to last ≥3 weeks and included four core components: supervised physical training, nutritional optimization, psychological support, and aid in alcohol and smoking cessation. Additionally, patients were screened for the presence of anaemia and dysregulated glucose levels.

#### Physical training

The exercise programme consisted of three 60-min sessions per week (with each session on a different day) supervised by a local (extramural) physiotherapist to whom the patient was referred after screening. These sessions incorporated both high-intensity interval training (HIIT) and resistance exercise training. The HIIT protocol included 28 min of alternating intervals: 4 min at high intensity (90% of estimated peak oxygen consumption (VO_2_)) followed by 3 min at low intensity (30% of estimated peak VO_2_). Peak VO_2_ was estimated using the Steep Ramp Test (SRT), which was performed at baseline. Resistance exercise targeted major muscle groups through a standardized set of six exercises: leg press, chest press, abdominal crunch, low row, lateral pulldown, and step-up. Initial loads were set at 65% of the estimated one-repetition maximum (1RM), which was determined at baseline, and loads progressively increased by 5% each week. On the other 4 days of the week, patients were instructed to complete 60 min of moderate-intensity aerobic exercise.

#### Nutritional optimization

Nutritional support was provided by a registered (intramural) clinical dietitian during face-to-face nutritional counselling sessions, including personalized dietary advice and focusing on achieving sufficient protein, energy, and micronutrient intake throughout the prehabilitation interval. The goal was a daily protein intake of ≥1.5 g per kg body weight. Energy requirements were estimated using the WHO formula, with an additional 30% added to account for physical exercise. All patients received an initial consultation with a standard follow-up, and additional consultation if needed. To support dietary goals, all patients were provided with high-quality whey protein shakes (30 g protein and 20 µg vitamin D per serving) and a daily multivitamin supplement covering 50% of the recommended daily intake. Protein shakes consisted of ten servings per week (7 daily servings and 1 serving after each supervised training session).

#### Psychological support

Patients who scored ≥15 on the Hospital Anxiety and Depression Scale (HADS) and agreed to receive psychological support were referred to a psychologist for help with surgery-related anxiety and coping. This cut-off has been shown to demonstrate good diagnostic sensitivity and specificity for syndromal depression in patients with cancer^16^.

#### Alcohol and smoking cessation

Additionally, patients who smoked were invited to participate in a structured smoking cessation programme (SineFuma). All participants were instructed to refrain from alcohol during the preoperative interval.

### Control group

Patients in the control interval received standard preoperative care according to Radboudumc guidelines, which varied by clinical pathway and did not include multimodal prehabilitation. The standard approach aligns with national Dutch guidelines, including ERAS protocols^17^.

### Study outcomes and data collection

The primary outcome was the incidence and severity of postoperative complications within 30 days, with assessment of Clavien–Dindo (CD) grade ≥II complications and the dichotomized Comprehensive Complication Index (CCI)^18^. The CCI was calculated by summing all complications graded according to CD, offering a cumulative measure of complication burden^19^. As the CCI distribution was highly skewed, the score was dichotomized on the upper quartile of the CCI distribution to facilitate analysis^20^, which corresponded to 22.6 for the total cohort and 29.6 for the high-risk GI oncological surgery subgroup. This approach was decided upon after inspecting the distribution, but before statistical modelling.

Postoperative complications within 30 days after surgery were extracted from the electronic medical records and reviewed by multiple assessors with a medical background. Regular consensus meetings with L.D.D. and B.v.d.H. were held to resolve uncertainties regarding CD grading and discrepancies were resolved using the extended CD classification^21^. Blinding of the assessors was not feasible, as group allocation could be inferred from the date of surgery. To ensure data quality, a random sample of 25 patients per assessor was re-evaluated by the two most experienced assessors, who were blinded with regard to the first evaluation (L.D.D. and B.v.d.H.); if discrepancies were found for at least five patients (20%), the entire set of patients assessed by that reviewer was re-examined and rescored by L.D.D. and B.v.d.H.

The secondary outcome was the length of hospital stay in days, obtained from the electronic medical records.

The covariables age, sex, ASA grade, preoperative haemoglobin (Hb) level, and neoadjuvant treatment were retrieved from the electronic medical records. Exercise session attendance was documented by local physiotherapists. Adherence to the nutritional component was based on patient-reported intake of protein shakes and multivitamin supplements, as recorded through questionnaires. Adherence to the psychological and smoking cessation components was recorded as the proportion of patients referred to a medical psychologist and the proportion of patients who participated in the smoking cessation programme respectively.

### Sample size

The sample size was based on simulations described in the published study protocol^22^. Briefly, the study was powered to detect a 20% relative reduction in CD grade ≥II complications using a log-binomial model accounting for clinical pathway and time effects.

### Statistical analysis

All statistical analyses were performed using SPSS^®^ (IBM, Armonk, NY, USA; version 29). Descriptive analyses were conducted for the primary outcome and covariables. Categorical variables are presented as *n* (%) and continuous variables are presented as mean(s.d.) or median (interquartile range (i.q.r.)) for skewed distributions.

The effects of multimodal prehabilitation on complication risk (CD grade ≥II) and dichotomized CCI score were analysed using a generalized linear model with a log link and binomial error distribution, resulting in risk ratios and corresponding 95% confidence intervals. The effect of multimodal prehabilitation on the length of hospital stay was analysed using a generalized linear model with a log link and negative binomial error distribution, resulting in incidence rate ratios and corresponding 95% confidence intervals. Clustering of patients within clinical pathways was accounted for by including clinical pathway as a fixed effect in all models, together with time of inclusion to adjust for temporal trends across the stepped implementation interval. The covariables age, sex, ASA grade, preoperative Hb level, and neoadjuvant treatment were also included as fixed effects in some models.

All eligible patients within each interval were included in the primary analyses, regardless of individual adherence to prehabilitation components. Secondary adherence-based analyses were performed for patients who attended at least nine supervised exercise sessions, in accordance with the study protocol. All analyses were performed for the total cohort and for the GI oncological surgery subgroup. All hypothesis tests were two-sided and *P* < 0.050 was considered statistically significant.

## Results

**Fig. 2:**
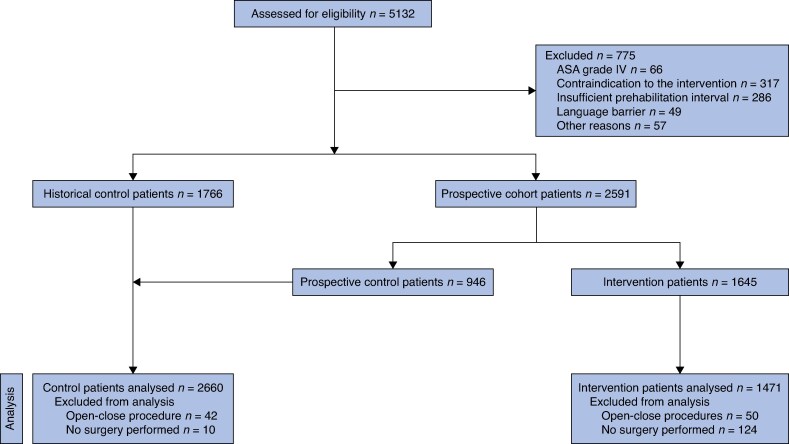
Study flow chart

Of the 5132 patients screened, 775 (15.1%) were excluded, mainly because of a contraindication to the intervention, an insufficient prehabilitation interval, or an ASA grade of IV (*Fig. 2*). Of the remaining 4357 patients scheduled for elective surgery, 2660 were analysed in the control group and 1471 were analysed in the intervention group after exclusion of patients who underwent open-close procedures or when surgery was ultimately not performed (*Fig. 2*). Recruitment was stopped because sufficient patients were included. Including more patients would not have contributed to the intended power of the study.

### Patient characteristics

The mean(s.d.) age of patients was 64.4(12.4) years, 44.6% were male, and 59.6% had an ASA grade of II (*Table 1*). Neoadjuvant treatment was more frequently administered in the prehabilitation group. Because of the stepped-wedge design, the distribution of patients across clinical pathways differed between groups. The proportion of patients who underwent surgery for rectal cancer (6.3% *versus* 2.0%), liver cancer or metastases (of colorectal origin) (8.8% *versus* 4.7%), and pancreatobiliary cancer (8.1% *versus* 5.8%) was higher in the prehabilitation group than in the control group and the proportion of patients who underwent surgery for hip arthrosis (14.1% *versus* 9.0%) and bladder cancer (9.8% *versus* 4.6%) was lower. Patient characteristics for the GI oncological surgery subgroup are also available in *Table 1*.

**Table 1:** Patient characteristics—total cohort and GI oncological surgery subgroup

	Total cohort		GI oncological surgery subgroup	
	Control group (*n* = 2660)	Prehabilitation group (*n* = 1471)	Control group (*n* = 590)	Prehabilitation group (*n* = 640)
Age (years), mean(s.d.)	64.6(12.4)	64.0(12.3)	64.9(11.2)	63.6(12.0)
Male	1144 (43.0)	697 (47.4)	343 (58.1)	400 (62.5)
**ASA grade**				
I	190 (7.2)	74 (5.0)	25 (4.2)	11 (1.7)
II	1577 (59.4)	886 (60.3)	360 (61.1)	407 (63.6)
III	890 (33.5)	510 (34.7)	204 (34.6)	222 (34.7)
**Neoadjuvant treatment**				
None	2273 (85.5)	1146 (78.0)	365 (61.9)	374 (58.4)
Chemotherapy	222 (8.3)	133 (9.0)	75 (12.7)	80 (12.5)
Radiotherapy	16 (0.6)	17 (1.2)	8 (1.4)	15 (2.3)
Chemoradiotherapy	139 (5.2)	165 (11.2)	137 (23.2)	165 (25.8)
Immunotherapy	10 (0.4)	9 (0.6)	5 (0.8)	5 (0.8)
Preoperative Hb level (mmol/L), mean(s.d.)	8.2(1.0)	8.2(1.0)	8.1(1.0)	8.2(1.0)
**Clinical pathway**				
Colon cancer	35 (1.3)	39 (2.7)	35 (5.9)	39 (6.1)
Rectal cancer	52 (2.0)	92 (6.3)	52 (8.8)	92 (14.4)
Liver cancer or metastases (of colorectal origin)	124 (4.7)	130 (8.8)	124 (21.0)	130 (20.3)
(Retro)peritoneal malignancies	82 (3.1)	111 (7.5)	82 (13.9)	111 (17.3)
Oesophageal cancer	143 (5.4)	149 (10.1)	143 (24.2)	149 (23.3)
Pancreatobiliary cancer	154 (5.8)	119 (8.1)	154 (26.1)	119 (18.6)
Abdominal aortic aneurysm (open repair)	25 (0.9)	21 (1.4)	NA	NA
Abdominal aortic aneurysm (endovascular repair)	49 (4.8)	35 (2.4)	NA	NA
Oral cancer	128 (4.8)	82 (5.6)	NA	NA
Laryngeal cancer	36 (1.4)	13 (0.9)	NA	NA
Supratentorial meningioma	123 (4.6)	68 (4.6)	NA	NA
Autologous breast reconstruction (after breast cancer)	204 (7.7)	93 (6.3)	NA	NA
Endometrial cancer	123 (4.6)	74 (5.0)	NA	NA
Ovarian cancer	151 (5.7)	60 (4.1)	NA	NA
Vulvar cancer	138 (5.2)	50 (3.4)	NA	NA
Hip arthrosis	375 (14.1)	132 (9.0)	NA	NA
Hip arthroplasty failure	155 (5.8)	49 (3.3)	NA	NA
Knee arthroplasty failure	76 (2.9)	22 (1.5)	NA	NA
Renal cancer	227 (8.5)	65 (4.4)	NA	NA
Bladder cancer	260 (9.8)	67 (4.6)	NA	NA

Values are *n* (%) unless otherwise indicated. GI, gastrointestinal; Hb, haemoglobin; NA, not applicable.

### Completion of the multimodal protocol items

Patients in the prehabilitation group completed a median of 5 (i.q.r. 0–8) supervised exercise sessions (*Table 2*). In total, 24.9% of patients attended at least nine exercise sessions, 69.6% reported daily consumption of protein shakes, 72.2% reported daily consumption of vitamin supplements, and 11.3% were referred to a medical psychologist. Of the 149 active smokers, 71 (47.6%) participated in the smoking cessation programme. During exercise, one patient experienced a vasovagal reaction and was subsequently assessed in the emergency department.

**Table 2.** Adherence to multimodal prehabilitation (*n* = 1471 for the prehabilitation group)

	Values
**Exercise sessions, median (i.q.r.)**	5 (0–8)
0–8 exercise sessions	1104 (75.1)
≥9 exercise sessions	367 (24.9)
**Protein shakes on a daily basis**	
Yes	1024 (69.6)
No	447 (30.4)
**Vitamin supplements on a daily basis**	
Yes	1062 (72.2)
No	409 (27.8)
**Referral to a medical psychologist**	
Yes	166 (11.3)
No	1305 (88.7)
Participation in the smoking cessation programme*	
Yes	71 (47.6)
No	78 (52.4)

Values are *n* (%) unless otherwise indicated. *Based on 149 active smokers.

### Outcomes of multimodal prehabilitation

At 30 days after surgery, a postoperative complication of any grade occurred in 1252 patients (47.1%) in the control group and 748 patients (50.8%) in the prehabilitation group (*Table 3*). The number of patients with CD grade ≥II complications was 981 (36.9%) in the control group and 596 (40.5%) in the prehabilitation group. The risk ratio was not statistically significant (adjusted risk ratio 1.02 (95% c.i. 0.90 to 1.16)). The proportion of patients with a CCI >22.6 was 23.7% in the control group and 28.1% in the prehabilitation group (adjusted risk ratio 1.03 (95% c.i. 0.86 to 1.23)). The median length of hospital stay was 5 (i.q.r. 3–9) in the control group and 6 (i.q.r. 3–10) in the intervention group (adjusted incidence rate ratio 1.04 (95% c.i. 0.92 to 1.18)). Adherence-based analyses (attendance of ≥9 exercise sessions) yielded comparable results.

**Table 3:** Postoperative complications and length of hospital stay—total cohort and GI oncological surgery subgroup

	Control group	Prehabilitation group	Risk ratio (95% c.i.) for postoperative complications and incidence rate ratio (95% c.i.) for length of hospital stay*	Risk ratio (95% c.i.) for postoperative complications and incidence rate ratio (95% c.i.) for length of hospital stay†
**Total cohort (*n* = 2660 for the control group and *n* = 1471 for the prehabilitation group)**				
Postoperative complications				
Any complication	1252 (47.1)	748 (50.8)	NA	NA
CD grade ≥II complications	981 (36.9)	596 (40.5)	1.01 (0.89,1.15)	1.02 (0.90,1.16)
CD grade ≥III complications	311 (11.7)	203 (13.8)	NA	NA
CD grade ≥IV complications	50 (1.9)	47 (3.2)	NA	NA
CD grade ≥V complications	19 (0.7)	15 (1.0)	NA	NA
CCI >22.6	631 (23.7)	413 (28.1)	1.00 (0.83,1.20)	1.03 (0.86,1.23)
Length of hospital stay (days), median (i.q.r.)	5 (3–9)	6 (3–10)	1.03 (0.91,1.16)	1.04 (0.92,1.18)
**GI oncological surgery subgroup (*n* = 590 for the control group and *n* = 640 for the prehabilitation group)**				
Postoperative complications				
Any complication	371 (62.9)	395 (61.7)	NA	NA
CD grade ≥II complications	340 (57.6)	330 (51.6)	0.90 (0.74,1.09)	0.91 (0.75,1.10)
CCI >29.6	139 (23.6)	142 (22.2)	0.90 (0.68,1.20)	0.95 (0.72,1.26)
Length of hospital stay (days), median (i.q.r.)	8 (6–13)	8 (6–11)	0.98 (0.78,1.23)	0.99 (0.79,1.24)

Values are *n* (%) unless otherwise indicated. *Models adjusted for clinical pathway and time of inclusion. †Models adjusted for clinical pathway, time of inclusion, age, sex, ASA grade, preoperative Hb level, and neoadjuvant treatment. GI, gastrointestinal; NA, not applicable; CD, Clavien–Dindo; CCI, Comprehensive Complication Index; Hb, haemoglobin.

In the GI oncological surgery subgroup, 340 patients (57.6%) in the control group and 330 patients (51.6%) in the prehabilitation group experienced a CD grade ≥II complication (*Table 3*). The risk ratio was not statistically different (adjusted risk ratio 0.91 (95% c.i. 0.75 to 1.10)). The proportion of patients with a CCI >29.6 was 23.6% in the control group and 22.2% in the prehabilitation group (adjusted risk ratio 0.95 (95% c.i. 0.72 to 1.26)). The median length of hospital stay was 8 days in both the control group (median of 8 (i.q.r. 6–13) days) and the intervention group (median of 8 (i.q.r. 6–11) days) (adjusted incidence rate ratio 0.99 (95% c.i. 0.79 to 1.24)). Adherence-based analyses did not reveal substantial differences.

## Discussion

In this single-centre, prospective stepped-wedge implementation study, we evaluated the hospital-wide effect of a multimodal prehabilitation programme across 20 surgical clinical pathways. Overall, the intervention did not significantly reduce postoperative complications or length of hospital stay compared with the control group. As a secondary finding, in the high-risk GI oncological surgery subgroup, the relative reduction in CD grade ≥II complication risk was 9%, albeit not statistically significant.

The absence of a beneficial effect in the overall surgical population reflects ambiguity of the current evidence highlighted in previous systematic reviews^23,24^. Studies in urological and orthopaedic settings have not demonstrated reduced complication risk after prehabilitation, but did find improvements in preoperative functional capacity^25,26^. However, studies among patients undergoing major abdominal surgery, who are widely recognized as being at high risk, have reported significant reductions in postoperative complications^10,11,27^. The present study adds to the literature as it uniquely encompassed a broad range of surgical pathways, including patient populations with little or no existing evidence on the effectiveness of prehabilitation. Additionally, the 9% difference in relative CD grade ≥II complication rates among the GI oncological surgery subgroup suggests that the effect of prehabilitation may be larger for patients with an elevated baseline risk. Although this subgroup analysis was pre-planned, there may have been insufficient power to detect statistically significant effects. Pre-surgical risk may also vary within this specific high-risk population. Hence, future studies should identify high-risk populations and investigate whether they benefit from prehabilitation.

Several factors may have contributed to the absence of a significant effect on postoperative complications and length of hospital stay. Of note, while the stepped-wedge design enabled pragmatic, hospital-wide implementation, it also introduced considerable variation in patient engagement across clinical pathways. The results therefore reflect a real-world effectiveness in clinical practice. Although standardized protocols were employed, adherence to individual intervention components—particularly the supervised exercise sessions—varied considerably. This variation in adherence may originate from differences in patient motivation across clinical populations, logistical challenges such as limited preoperative time in certain clinical pathways, the presence of co-morbidities, and varying levels of physical fitness, exercise history, social support, and particularities of this specific hospital implementation^28^. Although the exercise programme and nutritional intervention were tailored to individual fitness and nutritional needs, further personalization and optimization of training intensity, frequency, or delivery method, as well as adding behavioural motivational interviewing, could enhance feasibility and effectiveness. Multimodal prehabilitation is a complex intervention that does not conform to a one-size-fits-all approach^29^. Nevertheless, also the adherence-based analysis in the present study did not show improved postoperative outcomes after prehabilitation, suggesting that also high adherence to the physical exercise component did not uniformly reduce postoperative complication risk and length of hospital stay.

Furthermore, as patients were referred to >400 different physiotherapists, there was likely substantial variation in intervention delivery. Similar to other clinical guidelines, the protocol provided limited guidance on how to tailor the intervention to patients with, for example, multiple co-morbidities^30^. Potential adaptations to the prehabilitation intervention to better suit individual patients may have inadvertently deviated from the exercise training principles, resulting in less effective exercise prescriptions^31^. Future research should closely monitor intervention fidelity and standardize strategies on how to tailor exercise to individual patients, especially to those with co-morbidities^32^.

Of note, while the stepped-wedge design had the advantage that all clinical pathways transitioned to prehabilitation, ensuring widespread implementation, the approach introduced challenges in comparing intervention and control groups. Although clinical pathway was adjusted for in the analyses of the present study, residual confounding due to unmeasured differences between groups may have been present. Moreover, although the intended implementation schedule was sequential, the timing of transition varied across pathways due to logistical and operational constraints, reflecting the realities of hospital-wide implementation and potentially introducing additional heterogeneity between groups. In addition, the non-randomized order of implementation represents a limitation of the study and may have contributed to imbalances between clinical pathways. The phased implementation of prehabilitation also occurred alongside ongoing changes in clinical practice. For instance, the implementation of prehabilitation in the rectal cancer clinical pathway coincided with the introduction of robotic surgery. While the impact of this shift is not known, it may be associated with a learning curve and a potential temporary rise in complications^33^. Moreover, surgical indications also evolved. For instance, in pancreatic surgery, neoadjuvant chemotherapy has enabled resection of more locally advanced tumours, further increasing baseline surgical risk^34^. This may have diluted the effect of prehabilitation on postoperative outcomes. Finally, some behavioural spillover between care trajectories cannot be ruled out entirely. However, by design, all patients received standard preoperative care according to Radboudumc guidelines in the control interval. This did not include structured multimodal prehabilitation. Once the intervention phase began, the entire care trajectory switched to the prehabilitation programme and all patients were offered this programme as part of routine care. Given this transition of entire care trajectories, the risk of contamination between care trajectories is small.

A key strength of the present study is the hospital-wide implementation, allowing evaluation of prehabilitation in 20 clinical pathways. This broad scope also allowed the assessment of both the real-world effectiveness and efficacy of the intervention. Although the overall impact on postoperative complications and length of hospital stay was limited in the overall surgical population, prehabilitation may offer other benefits, such as improved mental well-being, especially a greater sense of control over one’s health, and reduced pain, anxiety, and depression after surgery^35^. It should be noted, however, that psychological support was only offered to patients with higher levels of psychological distress, which may have resulted in undertreatment of some patients with milder symptoms, thereby underestimating the psychological impact of prehabilitation. Broadening access to psychological support may therefore represent a valuable opportunity to further optimize the prehabilitation programme. Future evaluations of effects on patient-reported outcome measures, collected for prospective cohorts, may provide additional insight into these benefits. Furthermore, prehabilitation, especially in combination with appropriate postoperative rehabilitation, may enhance physical capacity, thereby supporting recovery and promoting improved long-term quality of life in patients.

In the context of value-based healthcare, further research is warranted to explore these additional dimensions. At Radboudumc, the hospital-wide implementation of multimodal prehabilitation was guided by a strategic vision that extended beyond its clinical objectives. The hospital’s board of directors recognized the urgent need for scalable interventions that help patients adopt healthier lifestyles, particularly amid growing rates of physical inactivity, poor nutrition, smoking, and alcohol use. These behaviours are major contributors to chronic disease and place increasing pressure on already overburdened healthcare systems^9^. Multimodal prehabilitation was recognized as a promising entry point for long-term health promotion and sustainable change^3^. While clinical outcomes offer important insights into the effectiveness of prehabilitation, understanding how such interventions are implemented is equally critical. Programme fidelity, reach, and patient engagement are key factors that influence the real-world impact of prehabilitation. A separate process evaluation is currently underway to support interpretation of the findings, refine implementation strategies, and inform broader policy development.

## Collaborators

K. Allewijn (Radboudumc, Nijmegen, the Netherlands); M.G.A. van den Berg (Radboudumc, Nijmegen, the Netherlands); P.B. van den Boezem (Radboudumc, Nijmegen, the Netherlands); J.J. Bonenkamp (Radboudumc, Nijmegen, the Netherlands); A.J.A. Bremers (Radboudumc, Nijmegen, the Netherlands); M. Dirven (Radboudumc, Nijmegen, the Netherlands); S.A.W. van de Groes (Radboudumc, Nijmegen, the Netherlands); M. Groos (Radboudumc, Nijmegen, the Netherlands); A.G. van der Heijden (Radboudumc, Nijmegen, the Netherlands); J. Honings (Radboudumc, Nijmegen, the Netherlands); H. Jager-Wittenaar (Radboudumc, Nijmegen, the Netherlands); B.R. Klarenbeek (Radboudumc, Nijmegen, the Netherlands); J.M. van Koeveringe (Radboudumc, Nijmegen, the Netherlands); B.M. van der Kolk (Radboudumc, Nijmegen, the Netherlands); M. ter Laan (Radboudumc, Nijmegen, the Netherlands); S. Muselaers (Radboudumc, Nijmegen, the Netherlands); J.M.A. Pijnenborg (Radboudumc, Nijmegen, the Netherlands); P. Servaes (Radboudumc, Nijmegen, the Netherlands); D. Smits (Radboudumc, Nijmegen, the Netherlands); S. Teerenstra (Radboudumc, Nijmegen, the Netherlands); T. Verhoeven (Radboudumc, Nijmegen, the Netherlands); J.H.W. de Wilt (Radboudumc, Nijmegen, the Netherlands).

## Supplementary Material

znag013_Supplementary_Data

## Data Availability

The data sets generated and analysed for this study are not publicly available. Data will be made available upon reasonable request. Requests for access to the data from this study can be submitted via e-mail to luuk.drager@radboudumc.nl.
