# Supplementary material for: Effects of multimodal prehabilitation on surgery outcomes: prospective stepped-wedge, hospital-wide implementation study
Source: Br J Surg. 2026 Feb 17;113(3):znag013. doi: 10.1093/bjs/znag013 (PMC13017783; doi:10.1093/bjs/znag013)

**Effects of Multimodal Prehabilitation on High-impact Surgery Outcomes: a prospective stepped-wedge, hospital-wide implementation study**

Luuk D. Drager, MD ^a^, Femke Atsma, PhD ^b^, Dieuwke Strijker, PhD ^a^, Linda A.G. van Heusden-Scholtalbers, MSc ^c^, Monique J.M.D. van Asseldonk, MSc ^d^, Jonas Rosenstok, MA ^a^, Joost P.H. Seeger, PhD ^e^, Sjors Verlaan, PhD ^a,f^, Laurien M. Buffart, PhD ^g^, Prof Cornelis J.H.M. van Laarhoven, PhD ^a^, Baukje van den Heuvel, PhD ^a^ on behalf of the F4S PREHAB Collaborative Group

**Affiliations**

^a^ Department of Surgery, Radboud University Medical Centre, Nijmegen, the Netherlands

^b^ IQ Health Science Department, Radboud University Medical Centre, Nijmegen, the Netherlands

^c^ Department of Rehabilitation, Radboud University Medical Centre, Nijmegen, the Netherlands

^d^ Department of Gastroenterology and Hepatology, Dietetics, Radboud

University Medical Centre, Nijmegen, the Netherlands

^e^ HAN University of Applied Sciences, Nijmegen, the Netherlands

^f^ Department of Nutrition and Dietetics, Faculty of Health, Sport and Physical Activity, Amsterdam University of Applied Sciences, Amsterdam, the Netherlands

^g^ Department of Medical Biosciences, Radboud University Medical Centre, Nijmegen, the Netherlands

**Corresponding author:**

Luuk D. Drager, MD

Geert Grooteplein Zuid 10, 6525 GA Nijmegen

E-mail: [luuk.drager@radboudumc.nl](mailto:luuk.drager@radboudumc.nl)

ORCID ID: 0000-0003-3283-095X

**Supplementary Materials - Index**

| **Supplementary Appendices** |  |
| --- | --- |
| Diagnoses according to the International Classification of Diseases 11^th^ revision | *page 3* |
| Checklist of information to include when reporting a stepped wedge cluster trial (CONSORT) | *page 6* |
|  |  |
|  |  |

**Supplementary Methods**

**Diagnoses according to the International Classification of Diseases 11^th^ revision**

Colon cancer

2B90 – Malignant neoplasms of colon

Rectal cancer

2B91 – Malignant neoplasms of rectosigmoid junction

2B92 – Malignant neoplasms of rectum

Liver cancer or metastases (of colorectal origin)

2C12 – Malignant neoplasm of liver

2D80·1 – Malignant neoplasm metastasis in liver

(retro)peritoneal malignancies

2B58·0 – Leiomyosarcoma of retroperitoneum or peritoneum

2B59·1 – Liposarcoma of retroperitoneum or peritoneum

2D91 – Malignant neoplasm metastasis in peritoneum

Oesophageal cancer

2B70 – Malignant neoplasms of oesophagus

2B71 – Malignant neoplasms of oesophago-gastric junction

Pancreaticobiliary cancer

2C10 – Malignant neoplasm of pancreas

2C15 – Malignant neoplasms of biliary tract, distal bile duct

2C16 – Malignant neoplasms of ampulla of Vater

Abdominal aortic aneurysm

BD50·4Z Abdominal aortic aneurysm, without mention of perforation or rupture

Oral cancer

2B61 – Malignant neoplasms of base of tongue

2B62 – Malignant neoplasms of other or unspecified parts of tongue

2B64 – Malignant neoplasms of floor of mouth

2B65 – Malignant neoplasms of palate

2B66 – Malignant neoplasms of other or unspecified parts of mouth

2B6A – Malignant neoplasms of oropharynx

Laryngeal cancer

2C23 – Malignant neoplasms of larynx

Supratentorial meningioma

2A01·0 – Meningiomas

Autologous breast reconstruction (post breast cancer)

QF01·0 Acquired absence of breast

Endometrial cancer

2C76 – Malignant neoplasms of corpus uteri

Ovarian cancer

2C73 – Malignant neoplasms of ovary

Vulvar cancer

2C70 – Malignant neoplasms of vulva

Hip arthrosis

FA00 – Osteoarthritis of hip

Hip arthroplasty failure

QB51·7 – Presence of orthopaedic joint implants

Knee arthroplasty failure

QB51·7 – Presence of orthopaedic joint implants

Renal cancer

2C90 – Malignant neoplasms of kidney, except renal pelvis

2C91 – Malignant neoplasms of renal pelvis

2C92 – Malignant neoplasms of ureter

Bladder cancer

2C94 – Malignant neoplasms of bladder

**Checklist of information to include when reporting a stepped wedge cluster trial (CONSORT)**


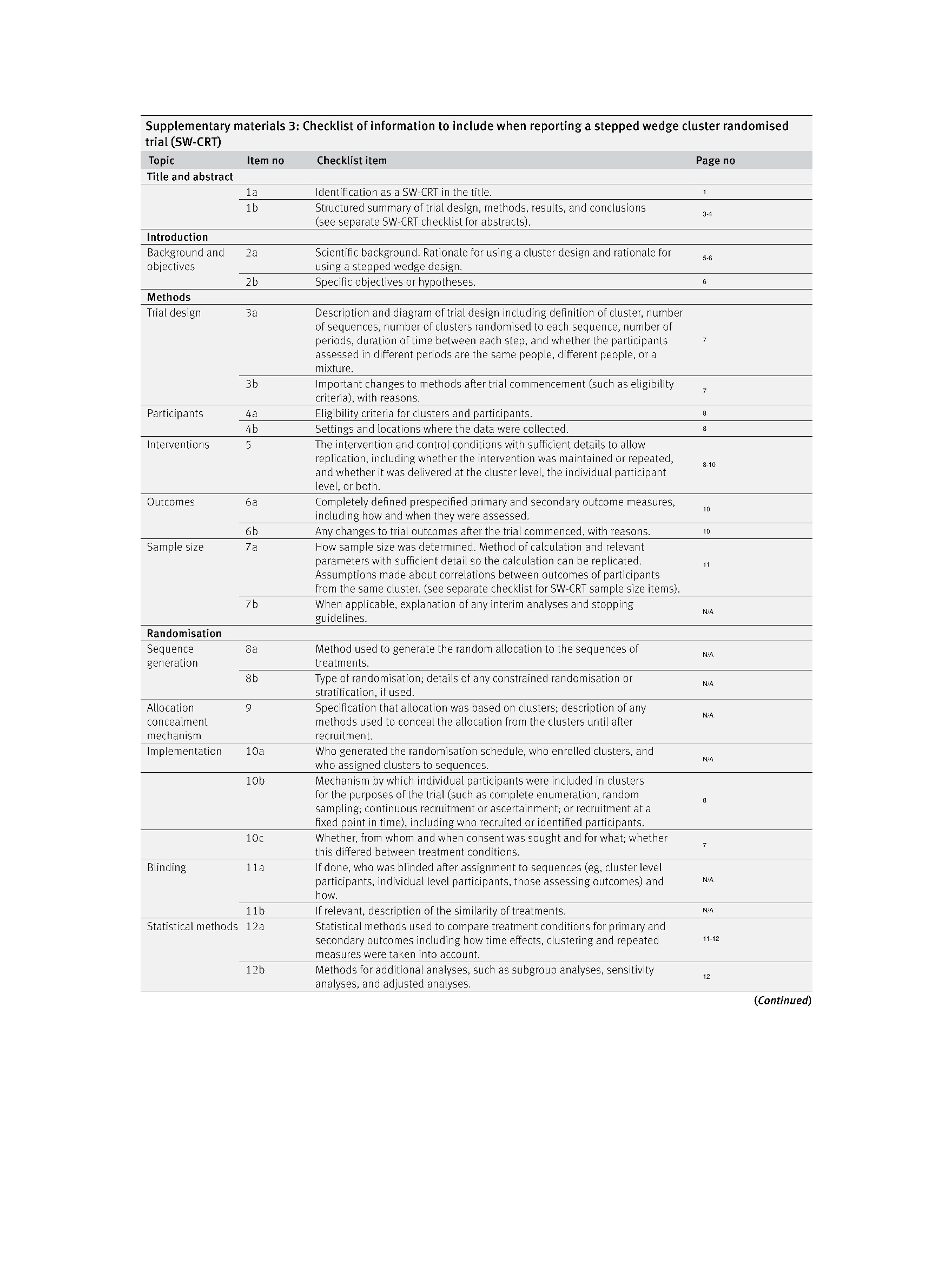


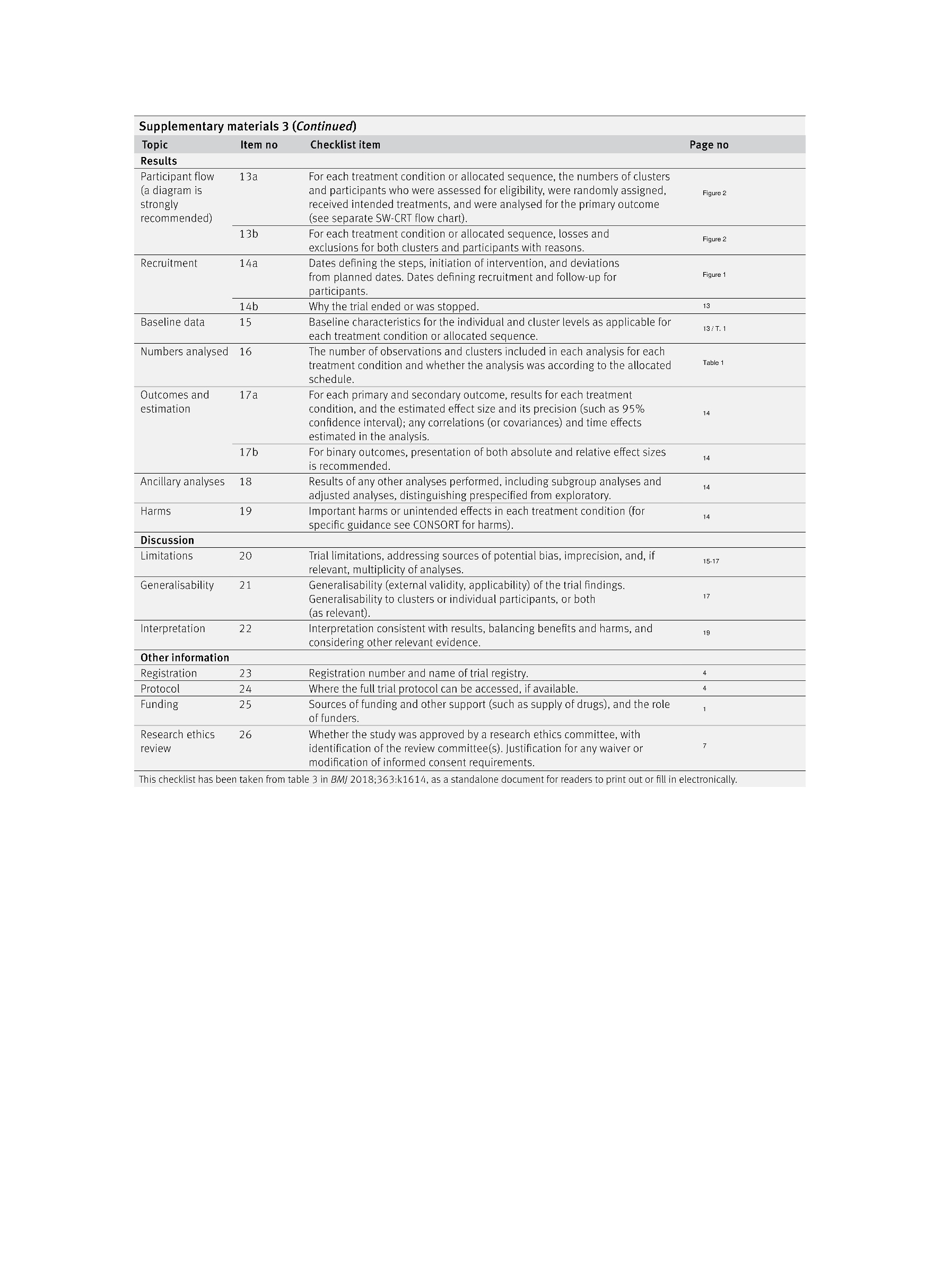

Supplement: znag013_Supplementary_Data [file znag013_supplementary_data.docx]
